# Adherence to the Mediterranean Diet and Its Association with LDL-Cholesterol and Subendocardial Viability Ratio in Individuals with Familial Hypercholesterolemia: A Cross-Sectional Study

**DOI:** 10.3390/nu18060919

**Published:** 2026-03-14

**Authors:** Nicoletta Miano, Sabrina Scilletta, Maurizio Di Marco, Stefania Capuccio, Marina Martedì, Marta Coppa, Norbert Tincu, Salvatore Carasi, Caterina Ippolito, Claudia Pistritto, Cecilia Di Stefano, Andrea Scarfia, Christian Pennisi, Giosiana Bosco, Francesco Di Giacomo Barbagallo, Antonino Di Pino, Salvatore Piro, Roberto Scicali

**Affiliations:** 1 Department of Clinical and Experimental Medicine, University of Catania, 95124 Catania, Italy; nicoletta.miano@gmail.com (N.M.); sabrinascilletta@gmail.com (S.S.); maurizio.dimarco@studium.unict.it (M.D.M.); stefania.cap@hotmail.com (S.C.); marinamartedi@gmail.com (M.M.); coppamarta@gmail.com (M.C.); norbert.tincu2008@gmail.com (N.T.); salvo.carasi@gmail.com (S.C.); caterinaippolito98@gmail.com (C.I.); claudiapistritto@hotmail.com (C.P.); ceciliadistefano@live.it (C.D.S.); dr.andreascarfia@gmail.com (A.S.); dr.christianpennisi@gmail.com (C.P.); giosiana.bosco@gmail.com (G.B.); fdigiacomobarbagallo@gmail.com (F.D.G.B.); antonino.dipino@unict.it (A.D.P.); roberto.scicali@unict.it (R.S.); 2 Department of Medicine and Surgery, “Kore” University of Enna, 94100 Enna, Italy

**Keywords:** familial hypercholesterolemia, mediterranean diet, subendocardial viability ratio, cardiovascular risk

## Abstract

**Background/Objectives**: An intensive lipid-lowering therapy is needed in familial hypercholesterolemia (FH) subjects; however, the adherence to the Mediterranean diet (MD) and its effects have not been fully evaluated in FH subjects. This study aimed to evaluate the impact of the MD on metabolic and vascular profiles in FH subjects. **Methods**: In this cross-sectional study 253 genetically confirmed FH subjects were included. Adherence to MD was assessed by the validated Pyramid-based MD Score (PyrMDS) and FH subjects were stratified according to the tertiles of PyrMDSs (low, intermediate, and high), with a higher score indicating higher adherence to MD. The lipid profile as well as the subendocardial viability ratio (SEVR), an indirect measure of myocardial perfusion, were assessed in all FH subjects. **Results**: Compared to the low-PyrMDS group, FH subjects with a high MD adherence showed lower levels of low-density lipoprotein cholesterol (LDL-C) (149.7 ± 71.4 vs. 176.7 ± 77.4 mg/dL, *p* = 0.006). After accounting for lipid-lowering therapies, smoking habit, and arterial hypertension, individuals in the high-PyrMDS group showed higher SEVR than those in the intermediate- and low-PyrMDS groups (167 ± 3.51 [standard error—SE] vs. 150 ± 5.03 [SE] vs. 148 ± 3.75 [SE], all *p* < 0.01). After adjusting for age, sex, and lipid-lowering therapies, PyrMDS was independently associated with LDL-C (β = −0.11, *p* = 0.03). **Conclusions**: Greater adherence to the MD was associated with more favorable metabolic and vascular profiles in FH subjects independent of lipid-lowering therapies. This suggests that MD adherence should be actively promoted in clinical practice alongside pharmacological interventions.

## 1. Introduction

Familial hypercholesterolemia (FH) is a monogenic autosomal dominant metabolic disorder, marked by high low-density lipoprotein cholesterol (LDL-C) leading to early atherosclerotic cardiovascular disease onset [1]. This condition represents an important clinical issue because it is often underdiagnosed, although it has an estimated prevalence of about 1 to 250 for the heterozygous form [2]. Importantly, individuals with FH have up to 13-fold increased risk of coronary heart disease in comparison to people without it [3].

Lowering LDL-C levels is crucial to slowing the course of atherosclerosis progression in FH subjects [4], and intensive lipid-lowering strategies are required in these subjects [5]. However, lifestyle improvement is traditionally considered secondary to pharmacological treatment [6,7]. Indeed, it has been shown that FH subjects frequently adopt unhealthy eating behaviors because lifestyle modification is perceived as providing less benefit for LDL-C reduction than lipid-lowering therapies [8]. Nevertheless, European guidelines on dyslipidemia management recommend that FH subjects follow dietary patterns with proven cardiometabolic benefits in addition to pharmacological therapy [9,10]. Importantly, beyond modestly reducing LDL-C levels, a healthy diet may improve the overall cardiovascular risk profile by positively affecting hypertension, obesity, and type 2 diabetes, and by counteracting the progression of pro-atherogenic pathways—such as oxidative stress, vascular inflammation, and endothelial dysfunction—in FH subjects [11,12,13].

Within this context, the Mediterranean diet (MD), characterized by a high consumption of monounsaturated fatty acids derived from olive oil and foods derived from high-fiber plants, has been associated with a reduced incidence of cardiovascular events compared with other dietary patterns in dysmetabolic populations [14].

Moreover, data from meta-analyses have shown that the MD is associated with a reduction in total or LDL cholesterol in various clinical settings, such as diabetes and metabolic dysfunction-associated steatotic liver disease [15,16]. Interestingly, a randomized controlled trial in a high-cardiovascular-risk population highlighted that an MD supplemented with nuts shifts lipoprotein subfractions toward a less atherogenic pattern, with a reduction in small LDL-C particles and an increase in large high-density lipoprotein cholesterol (HDL-C) particles. Among the components of the MD, water-soluble fibers and nuts have been shown to reduce LDL-C [17,18].

In FH population, adherence to the MD has also been independently associated with a more favorable inflammatory profile, as shown in a cohort of molecularly confirmed FH individuals from Brazil and Spain [7]. Given the role of inflammation in the development of subclinical atherosclerosis in FH, bioactive dietary components such as phytosterols—known to exert anti-inflammatory effects through cyclooxygenase-2 inhibition—may contribute to modulating cardiovascular risk beyond lipid lowering alone [19,20,21,22].

Despite these benefits, adherence to healthy dietary pattern remains challenging in clinical practice, and data from a systematic review reported that the vast majority of the 50 included studied documented only low or moderate adherence to the MD in Mediterranean countries [23], highlighting the gap between recommendations and real-world practice.

Although the cardiovascular benefits of the MD are well documented in the general population, evidence in genetically confirmed FH subjects remains limited, particularly regarding vascular functional markers beyond lipid levels. To date, most studies in FH have focused on structural or peripheral vascular markers (such as carotid intima–media thickness or arterial stiffness), while markers reflecting myocardial perfusion and the balance between oxygen supply and demand at the subendocardial level have been largely overlooked.

In this context, the subendocardial viability ratio (SEVR) represents an integrative functional marker of coronary microvascular perfusion and myocardial oxygen supply–demand balance, providing complementary information to traditional vascular indices. However, data on SEVR in individuals with genetically confirmed FH are currently lacking, and no previous studies have specifically explored its relationship with dietary patterns in this high-risk population.

Therefore, the present study aimed to evaluate adherence to MD using the Pyramid-based Mediterranean Diet Score (PyrMDS) in a cohort of individuals with genetically confirmed FH and its impact on metabolic and vascular profiles, as measured by SEVR. By focusing on SEVR as a functional vascular marker, this study seeks to provide novel insights into the potential incremental value of dietary adherence on myocardial perfusion-related vascular health beyond lipid lowering alone. These findings may provide clinically relevant insights to support the integration of structured dietary counseling alongside pharmacological therapy in the management of FH.

## 2. Materials and Methods

### 2.1. Experimental Approach

In this cross-sectional observational study, individuals with genetically confirmed FH were enrolled from the Lipid Center of University Hospital, Catania, Italy, from November 2023 to December 2025. This is a tertiary care center, specialized in the screening, diagnosis and management of dyslipidemias [24].

The protocol of this study was examined and approved by the local ethics committee (Comitato Etico Catania 2, Piazza Santa Maria di Gesù n° 5, Catania, Italy, protocol n. 67/C.E.L. 11 October 2023). Informed consent was obtained from each participant.

### 2.2. Study Subjects

Individuals with genetically confirmed FH aged between 18 and 70 years were eligible for inclusion. Exclusion criteria were the presence of hematological diseases, malignancies, thyroid disorders, chronic liver diseases, acute infections, autoimmune diseases, chronic inflammatory conditions, or treatment with systemic glucocorticoids within the previous three months. All participants underwent a complete physical exam, a review of their clinical history, as well as biochemical analyses after overnight fasting, and vascular profile evaluation by assessment of the subendocardial viability ratio (SEVR) as an indirect measure of left ventricular myocardial perfusion during workload [25]. Arterial hypertension was defined as a brachial blood pressure of ≥140 mm Hg systolic and/or ≥90 mm Hg diastolic measured on at least two separate occasions, or the current use of antihypertensive medication [26]. Lipid-lowering therapy was defined as the daily use of any of the following agents: statins, ezetimibe, or a proprotein convertase subtilisin/kexin type 9 (PCSK9) inhibitor (alirocumab, evolocumab, or inclisiran) [24]. Body weight and height were measured, and body mass index (BMI) was calculated as weight divided by the squared value of height (kg/m^2^). Diabetes mellitus was defined as fasting plasma glucose ≥ 126 mg/dL on two consecutive readings and/or glycated hemoglobin (HbA1c) ≥ 6.5% or the use of anti-diabetic medications [27]. Smoking habits were divided into either current smoking (defined as any cigarette in the last month) or not [28]. LDL-C was calculated using the Friedwald formula.

### 2.3. Nutritional Assessment

Nutritional data were collected by administering a previously validated form of the Pyramid-based Mediterranean Diet Score (PyrMDS) [29]. The PyrMDS questionnaire was administered through personal interviews conducted by trained investigators. Participants were provided with standardized instructions regarding questionnaire completion and portion size estimation prior to administration. All interviewers underwent specific training and followed a standardized protocol to ensure uniform data collection and to reduce potential misinterpretation and interviewer-related bias. The PyrMDS is composed of 15 elements, and a different weight is given for both underconsumption and overconsumption. This score is the only one that adopts a unique graduated scoring system that is different for specific food groups: a threshold for excessive consumption of unhealthy foods (red and processed meat, sweets), a bell curve for alcohol, a partial drop in score for excessive consumption of fruit, nuts, cereals, dairy products, white meat and eggs and no negative effect of excessive consumption of vegetables, legumes and fish.

Specifically, suboptimal consumption of legumes and vegetables (high-frequency healthy foods) is scored with values from 0 to 0.99 in proportion to portions (actual/optimal). The same applies to foods to be consumed in moderation (fruit, nuts, cereals, dairy products, white meat, eggs) for which excessive consumption (twice the value of the midpoint of the recommended intake) is penalized with a score of 0.5. Excessive consumption of low-frequency foods (red and processed meat, potatoes, sweets) is penalized similarly to foods to be consumed in moderation. For alcohol, excessive consumption has a score of 0, while no consumption has a score of 0.5.

### 2.4. Study Groups

Study population was divided into three groups according to the PyrMDS tertiles in order to explore potential dose–response relationships between adherence to the Mediterranean diet and cardiometabolic outcomes.

The study groups were defined as follows: low level of adherence to the Mediterranean diet corresponding to the low-PyrMDS group (<6.64 score, 83 subjects), moderate level of adherence corresponding to the intermediate-PyrMDS group (6.64–8.64 score, 84 subjects), and high level of adherence to the Mediterranean diet corresponding to the high-PyrMDS group (≥8.65 score, 86 subjects).

### 2.5. Subendocardial Viability Ratio (SEVR)

Buckberg SEVR was calculated as the area of diastole divided by the area of systole during one cardiac cycle in the aorta, by analyzing aortic wave forms measured from the right radial artery using applanation tonometry with a Millar tonometer (SPC-301; Millar Instruments, Houston, TX, USA), as previously described [30]. All measurements were conducted by a single investigator with the subject in the supine position. Data were recorded directly on a desktop computer and analyzed using SphygmoCorCvMS (AtCor Medical, Sydney, Australia) [31].

### 2.6. Statistical Analyses

The sample size was calculated based on SEVR using a level of significance (α) set to 5% and a power (1 − β) set to 80%. Based on previous data in individuals with metabolic syndrome, a standard error (SE) of 3.3 was considered, corresponding to an estimated standard deviation (SD) of 26.4 [32]. A difference of 20 units in SEVR was considered clinically meaningful, corresponding to a Cohen’s d of 0.76. The estimated sample size was 25 subjects per group for ANOVA comparisons. To account for adjustment for covariates, the sample size was increased by 20 participants per group, resulting in 45 subjects per group. Therefore, the total sample size was 135 participants. The actual sample size in each PyrMDS tertile exceeded this threshold.

The distributional characteristics of each variable, including normality, were assessed using the Kolmogorov–Smirnov test. Data are reported as mean ± SD for continuous parametric variables, median (IQR) for continuous non-parametric variables, and frequency (percentage) for categorical variables. When necessary, continuous non-parametric variables—such as triglycerides (TG), high-sensitivity C-reactive protein (hs-CRP), lipoprotein(a) [Lp(a)]—were logarithmically transformed for statistical analysis to reduce skewness. Group comparisons were performed using ANOVA test for continuous variables and the Chi-square test for categorical variables. ANCOVA test was employed to compare the value of SEVR adjusted for lipid-lowering therapies, smoking habit, and arterial hypertension. The result of ANCOVA was expressed as mean ± SE. To evaluate the impact of MD adherence on LDL-C, a simple regression analysis, as well as a multivariate regression analysis adjusted for age, sex, and lipid-lowering therapies, was performed. In addition, prespecified subgroup analyses were performed by stratifying participants according to PCSK9-i use (yes/no) to explore whether the association between PyrMDS and LDL-C differed according to treatment with highly potent lipid-lowering agents. In each subgroup, multivariable linear regression models adjusted for age, sex, and lipid-lowering therapy were fitted. Given the limited number of patients treated with PCSK9-i, these subgroup analyses were considered exploratory.

Multicollinearity was excluded prior to multivariate analyses by estimating a variance inflation factor < 2. Statistical analyses were performed, and figures were generated using R 4.5.2 (R core team (Vienna, Austria)) and STATA 14 (StataCorp (College Station, TX, USA)). All tests were two-sided and a *p* < 0.05 was considered significant.

## 3. Results

### 3.1. General Characteristics and Lipid Profile of the Study Population

A total of 318 subjects were evaluated; of these, 253 FH subjects satisfied the inclusion criteria and participated in the study.

Clinical and biochemical characteristics of the study population are shown in Table 1. The mean age in the study groups ranged from 44.3 to 50.6 years, and the percentage of males ranged from 41.7 to 55.8%. The mean age at FH diagnosis ranged from 29.9 to 39.7 years. Patients with high PyrMDSs showed significantly lower levels of LDL-C in comparison to those with low PyrMDSs (149.7 ± 71.4 vs. 176.7± 77.4 mg/dL, *p* = 0.006) and intermediate PyrMDSs (149.7 ± 71.4 vs. 184.7 ± 80.7, *p* = 0.04). Lipoprotein(a) and TG were similar between the three groups.

**Figure 1:**
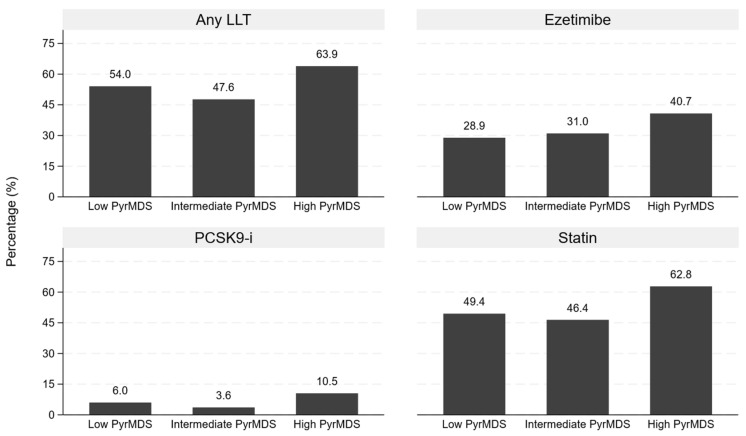
Lipid-lowering therapy in the study population. The bars represent the percentage of individuals treated with lipid-lowering therapies (LLT) in each tertile group of adherence to the Mediterranean diet assessed through Pyramid-based Mediterranean Diet Score (PyrMDS): ≤6.64 low PyrMDS, 6.64–8.64 intermediate PyrMDS, (≥8.65) high PyrMDS. PCSK9-i: proprotein convertase subtilisin/kexin type 9 inhibitor.

Figure 1 reports the data related to lipid-lowering therapy. The use of any lipid-lowering therapy ranged from 47.6 to 63.9%. However, the prevalence of PCSK9-i users was lower than 11% in all study groups.

### 3.2. SEVR in the Study Population

**Figure 2:**
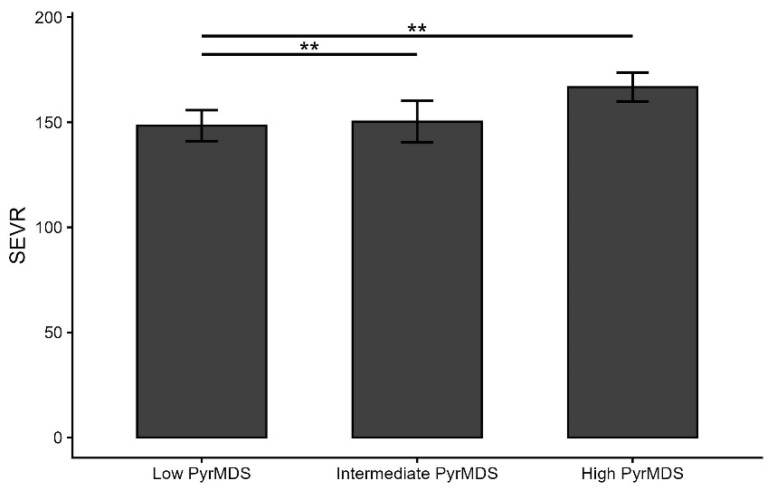
SEVR in the study population after adjusting for lipid-lowering therapies, smoking habit, and arterial hypertension. The figure shows the adjusted mean subendocardial viability ratio (SEVR) values for each tertile group of adherence to Mediterranean diet assessed through Pyramid-based Mediterranean Diet Score (PyrMDS): ≤6.64 low PyrMDS, 6.64–8.64 Intermediate PyrMDS, (≥8.65) High PyrMDS. All values were adjusted for lipid-lowering therapy using ANCOVA. Error bars represent 95% confidence intervals. ** *p* < 0.01.

As shown in Figure 2, after adjusting for lipid-lowering therapies, smoking habit, and arterial hypertension, individuals in the high-PyrMDS group showed a higher SEVR than those in intermediate- (167 ± 3.51 [SE] vs. 148 ± 3.75 [SE], *p* = 0.009) and low-PyrMDS groups (167 ± 3.51 [SE] vs. 150 ± 5.03 [SE], *p* = 0.0005).

### 3.3. Regression Analyses to Evaluate the Impact of PyrMDS on LDL-C

In simple regression analysis, LDL-C was associated with PyrMDS (r = −0.15, *p* = 0.03), and this association was also observed after adjusting for age, sex and lipid-lowering therapy (β = −0.11, *p* = 0.03), as shown in Table 2.

In subgroup analyses stratified by PCSK9 inhibitor use, a higher PyrMDS was significantly associated with lower LDL-C levels in patients not treated with PCSK9 inhibitors after adjustment for age, sex and lipid-lowering therapy (β = −8.34 mg/dL per point increase in PyrMDS, 95% CI −17.25 to −0.56, *p* = 0.049). In patients receiving PCSK9-i, the number of subjects was very limited (n = 15), resulting in unstable estimates and wide confidence intervals.

## 4. Discussion

In this study we assessed the adherence to the MD in a cohort of individuals with genetically confirmed FH and its impact on the metabolic and vascular profile. This was the first study evaluating MD adherence in this type of population by employing the PyrMDS, an easily applicable tool for clinical practice, and assessing its association with the vascular profile. We found that participants with a higher adherence to the MD exhibited lower LDL-C levels. Furthermore, they showed better myocardial perfusion, as assessed by SEVR, independent of the lipid-lowering therapy. Furthermore, lower LDL-C levels were associated with higher adherence to the MD independently from the confounders.

The finding of a more favorable lipid profile, particularly lower LDL-C levels, associated with adherence to the MD aligns with previous studies assessing the beneficial role of healthy dietary regimens in people with FH. A population-based Danish study, including more than 500 individuals with FH, highlighted that increasing levels of heart-healthy dietary adherence were associated with lower concentrations of LDL-C and non-HDL cholesterol, as well as a lower risk of ischemic heart disease, independently of lipid-lowering therapy [33]. Similarly, Antoniazzi et al. conducted a cross-sectional analysis of MD adherence in a cohort of 190 FH subjects from Brazil and Spain, reporting that higher adherence to the MD was associated with lower levels of LDL-C [9]. Notably, in our study the effect of the MD is independent of lipid-lowering therapies, highlighting the synergistic role of diet and pharmacological treatment in achieving lipid targets. Interestingly, individuals with higher adherence to the MD were also more likely to be receiving lipid-lowering therapies. This observation may reflect a greater overall health-oriented attitude, whereby individuals who are more attentive to lifestyle recommendations may also be more compliant with pharmacological treatments and other preventive behaviors. However, the association between MD adherence and both LDL-C and SEVR remained significant after adjustment for lipid-lowering therapies, supporting an independent relationship between dietary adherence and metabolic and vascular profiles. Nevertheless, residual confounding related to health-seeking behaviors and other unmeasured lifestyle factors cannot be completely excluded in the context of an observational cross-sectional design. A similar association between dietary adherence and pharmacological compliance has been reported in other clinical settings, such as type 2 diabetes [34].

In our study, the beneficial properties of the MD could explain the finding of a higher SEVR, reflecting better myocardial perfusion in people with higher adherence to the MD. SEVR is a clinically meaningful surrogate marker of myocardial oxygen supply–demand balance and subendocardial perfusion, and has been shown to be inversely associated with coronary microvascular dysfunction, arterial stiffness, and cardiovascular risk in different clinical settings. However, this index has not been thoroughly investigated in FH subjects, who are particularly prone to developing early cardiovascular disease and in whom it could allow the non-invasive and repeated assessment of myocardial perfusion. The observed association between greater adherence to the MD, lower LDL-C levels, and higher SEVR may reflect synergistic effects on coronary microcirculation and myocardial oxygen supply–demand balance. In individuals with FH, lifelong exposure to elevated LDL-C levels promotes endothelial dysfunction, increased arterial stiffness, and microvascular remodeling, which can impair subendocardial perfusion. By reducing LDL-C levels and providing bioactive compounds with anti-inflammatory and antioxidant properties the MD may improve endothelial function, enhance nitric oxide bioavailability, and reduce oxidative stress, collectively supporting better coronary microvascular function. This result is in line with previous findings of the protective role of the MD for the cardiovascular profile. Particularly, a meta-analysis of 16 studies showed an inverse association between the MD and arterial stiffness [35]. Moreover, Van de Laar et al., in a longitudinal study involving 373 apparently healthy participants, observed that those with stiffer carotid arteries had lower adherence to the MD during adolescence and early adulthood [36]. Yubero-Serrano et al., in the CORDIOPREV randomized controlled trial, found that participants randomized to follow the MD had better endothelial function assessed by flow-mediated dilation of the brachial artery, after 1 year from enrollment compared to those randomized to a low-fat diet [37]. Another recent study analyzed the relationship between dietary patterns and a score evaluating coronary morphology—the SYNTAX score—in 1121 patients undergoing coronary angiography from the INTERCATH study. The authors found that, after adjusting for confounders, higher adherence to the MD was associated with a reduced probability of medium/high-risk SYNTAX score, indicating a less complicated coronary disease [38].

Thus, our results highlight that non-pharmacological treatments, particularly dietary interventions, are associated with improved myocardial perfusion, as reflected by higher SEVR values—an association that has not been previously reported in the literature. For this reason, clinicians should actively promote adherence to the MD as an integrative strategy alongside pharmacological therapy. Furthermore, the assessment of adherence to healthy dietary patterns should represent a key component of outpatient visits.

There are various mechanisms which could explain this association. First of all, different compounds of the MD, such as water-soluble fiber and nuts, have been associated with a better lipid profile [17,18], as we found in our population. LDLC is a key factor in atherosclerosis and people with FH are exposed to high concentrations of these particles from birth [39,40]. Thus, the MD could contribute to reducing the LDL-C burden. In addition, the MD could exert beneficial effects on endothelial function through other mechanisms, such as increasing nitric oxide availability, decreasing P-selectin, and protecting against oxidative stress [41,42].

Our study has some limitations. First, the observational nature of the study does not allow us to establish a causal relationship between diet and lipid and vascular parameters. In this context, the possibility of reverse causality should be considered, as individuals with a more favorable metabolic and vascular profile or greater health awareness may be more likely to adhere to healthier dietary patterns, including the Mediterranean diet. In line with this issue, the risk of potential global adherence bias should be considered. Moreover, the use of PyrMDS to assess MD adherence relies on self-reported questionnaires, which may be subject to recall bias and social desirability bias. Future studies should integrate dietary assessment with objective biomarkers of dietary intake (e.g., plasma fatty acid profiles or other nutritional biomarkers) to validate self-reported adherence to the Mediterranean diet and strengthen the robustness of these findings. Furthermore, although SEVR is considered a reliable marker of myocardial perfusion, it represents an indirect parameter. In addition, despite adjustment for potential confounders in the statistical analyses, baseline differences in clinical characteristics between groups may have resulted in residual confounding. Finally, other parameters such as oxidative stress should be considered when assessing the beneficial role of the MD. Given these findings, further prospective interventional studies are needed to confirm the beneficial impact of the MD on the cardiometabolic profile in FH subjects.

## 5. Conclusions

Higher adherence to the MD was associated with a better lipid profile in individuals with FH, as well as with a higher SEVR, an indirect measure of myocardial perfusion. Thus, our study supports the role of the MD as an additional strategy to pharmacological therapy for managing FH. Promoting the adoption of this diet in FH patients could be useful for improving cardiometabolic control in this population, emphasizing the importance of an integrated approach between nutritional and pharmacological treatment.

## Figures and Tables

**Table 1:** Clinical and biometric characteristics of the study population stratified according to PyrMDS.

	Low PyrMDS (≤6.64) n = 83	Intermediate PyrMDS (6.64–8.64) n = 84	High PyrMDS (≥8.65) n = 86
Age (years)	44.3 ± 18.4	46.8 ± 17.3	50.6 ± 19.1
Men, n (%)	45 (54.2)	35 (41.7)	48 (55.8)
Age at diagnosis (years)	29.9 ± 15.1	31.6 ± 13.2	39.7 ± 12.9
BMI (kg/m^2^)	26.1 ± 3.9	25.6 ± 3.7	25.2 ± 3.7
Dutch score	15.1 ± 3.5	14.9 ± 3.0	15.0 ± 3.4
Active smokers, n (%)	21 (25.3)	17 (20.5)	18 (20.9)
Arterial hypertension, n (%)	8 (9.6)	10 (11.9)	21 (24.4)
Type 2 diabetes, n (%)	2 (2.4)	1 (1.2)	3 (3.5)
Total cholesterol (mg/dL)	247.0 ± 77.6	245.3 ± 75.4	211.5 ± 62.6 *#
Triglycerides (mg/dL)	81 (36–190)	89 (40–195)	78 (36–278)
LDL cholesterol (mg/dL)	176.7 ± 77.4	184.7 ± 80.7	149.7 ± 71.4 *#
HDL cholesterol (mg/dL)	53.1 ± 11.2	53.6 ± 14.3	53.2 ± 11.2
Hs-CRP (mg/dL)	0.12 (0.05–0.18)	0.08 (0.01–0.23)	0.10 (0.05–0.21)
Lp(a) (mg/dL)	21.1 (9.7–254)	13.9 (9.7–146)	16 (9.7–133)
HbA1c (%)	5.5 ± 0.3	5.4 ± 0.3	5.5 ± 0.3
FPG (mg/dL)	88.7 ± 14.1	86.5 ± 10.6	88.8 ± 10.4

Data are presented as mean ± SD, median (IQR), or percentage (absolute number). PyrMDS: Pyramid-based Mediterranean Diet Score. BMI: body mass index. LDL: low-density lipoprotein. HDL: high-density lipoprotein. Hs-CRP: high-sensitivity C-reactive protein. Lp(a): lipoprotein a. HbA1c: glycated hemoglobin. FPG: fasting plasma glucose. * *p* < 0.01 vs. low-PyrMDS group; *# p* < 0.01 vs. intermediate-PyrMDS group.

**Table 2:** Multiple regression analysis to evaluate the impact of PyrMDS on LDL-C.

Dependent Variable	LDL-C	
Independent Variables	Standardized β (95%CI)	*p* Value
Age (years)	0.01 (−0.10; 0.10)	0.86
Male sex	−0.07 (−0.17; −0.03)	0.16
Lipid-lowering therapies	−0.69 (−0.80; −0.59)	<0.0001
PyrMDS	−0.11 (−0.21; −0.01)	0.03

95%CI: 95% confidence interval. PyrMDS: Pyramid-based Mediterranean Diet Score. LDL-C: low-density lipoprotein cholesterol.

## Data Availability

The original contributions presented in this study are included in the article. Further inquiries can be directed to the corresponding author.

## Abbreviations

The following abbreviations are used in this manuscript:

FH	Familial Hypercholesterolemia
MD	Mediterranean Diet
HDL-C	High-Density Lipoprotein Cholesterol
hs-CRP	High-Sensitivity C-Reactive Protein
LDL-C	Low-Density Lipoprotein Cholesterol
PCSK9-i	Proprotein Convertase Subtilisin/Kexin type 9 Inhibitor
PyrMDS	Pyramid-based Mediterranean Diet Score
SEVR	Subendocardial Viability Ratio
TG	Triglycerides
